# Against the Environment. Problems in Society/Nature Relations

**DOI:** 10.3389/fsoc.2019.00029

**Published:** 2019-04-24

**Authors:** João Aldeia, Fátima Alves

**Affiliations:** ^1^Centre for Functional Ecology, University of Coimbra, Coimbra, Portugal; ^2^Department of Social Sciences and Management, Universidade Aberta, Lisbon, Portugal

**Keywords:** capitalism, environment, environmental crisis, nature, social sciences, society, sociology, western modernity

## Abstract

The dominant manners in which environmental issues have been framed by sociology are deeply problematic. Environmental sociology is still firmly rooted in the Cartesian separation of Society and Nature. This separation is one of the epistemic foundations of Western modernity—one which is inextricably linked to its capitalist, colonial, and patriarchal dimensions. This societal model reifies both humanity and nature as entities that exist in an undeniably anthropocentric cosmos in which the former is the only true actor. Anthropos makes himself and the world around him. He conquers, masters, and appropriates the non-human, turning it into the mere environment of his existence, there solely for his use. If sociology remains trapped in this paradigm it continues to be blind to the multiple space-time specific interrelations of life-elements through which heterogeneous and contingent ontologies of humans and extra-humans are enacted. If these processes of interconnection are not given due attention, the socioecological worlds in which we—human as well as others—live cannot be adequately understood. But misunderstandings are not the only issue at stake. When dealing with life-or-death phenomena such as climate change, to remain trapped inside the Society/Nature divide is to be fundamentally unable to contribute to world reenactments that do not oppress—or, potentially, extinguish—life, both human and extra-human. From the inside of Anthropos' relation to his environment the only way of conceiving current socioecological problems is by framing them in terms of an environmental crisis which could, hypothetically, be solved by the very same societal model that created it. But if the transformation of some of the world(s)' life-elements into the environment of the Human is part of the problem, then, socioecological issues cannot be adequately understood or addressed if they are framed as an environmental crisis. Instead, these problems need to be conceived as a crisis of Western modernity itself and of the kind of worlds that are possible and impossible to build within it.

## Introduction

Sociology studies interaction—specifically, it studies interactional distributions and enactments of power-knowledge and ontologies. That much still remains true. But there have been considerable changes—both within and outside the social sciences—since the times of Marx, Weber, Simmel, and other classical sociologists. The field's opening to the study of environmental issues has shattered many of what have historically been its epistemological and ontological foundations. As is the case of all of the social sciences and humanities, “thinking through the environment” deeply “unsettles” many of sociology's core assumptions^1^^,^^2^.

In order to be able to adequately understand “environmental issues”—and among them the very much urgent issue of climate change—sociology needs to move beyond the analytical exclusive focus on human interaction(s). There have been several proposals in this direction, but they still remain less than mainstream research stances (although it is arguable that this is less the case now than it was some decades ago). It is not possible to remain trapped inside the confines of what humans do with each other and expect to understand the myriad interrelations of human and extra-human life-elements^3^ of the world(s). To remain enclosed by an *a priori* defined privilege of human interaction is to stay incapable of seeing the true extent of the networks of life-elements that compose the socioecological phenomena that sociology studies. This does not mean that sociologists must become experts of all things, which would undoubtedly lead them to become experts of nothing. But there is a need to significantly widen the scope of the interrelations that we study. Humanity still has an important place in research, but the analytical focus must move from the intra-human to “the web of life,” to adopt Moore's (2016a,b) concept.

To be precise, the problem at hand is the Western modern paradigm of knowledge and practice in which sociology moves itself (i.e., the paradigm for whose constitution and maintenance it contributes). Among other things, this paradigm is patriarchal, colonial, capitalist, and anthropocentric. It institutionalizes forms of existence that enact specific types of human-nature relations where the second term is subordinated to the first (Santos, 1991, p. 14 *et seq*.; Santos, 2006, p. 91 *et seq*., p. 169 *et seq*; Plumwood, 1993; Lander, 2009; Latour, 2010; Moore, 2016a,b).

Given this, what is needed is not a calibration of research elements similar to the one that was done from the 1960s onward when, to accompany formal political decolonization happening in the Global South, sociology started opening its doors to the study of human worlds outside of the West (obviously, there where sociologists preoccupied with extra-Western phenomena before this date, but they were far from being the majority). To replicate this now is unsustainable because we are not dealing with more human elements to add to the mix. For some decades now, we have been facing an irruption of the extra-human into what has historically been a human-focused field of inquiry. It is not further human populations that are entering our field of vision but trees, and animals, and water, and gases, and rocks. As such, any attempt to merely add up a new element—the environment, nature, or whatever one chooses to call it—to our considerations simply does not work^4^.

If epistemological and ontological changes stop there, as they are prone to do, sociology is not doing anything very original. It is merely replicating the same Cartesian divide of Society vs. Nature that has, since its beginning, characterized it. For 200 years, sociologists have mainly dealt with this divide by focusing on just one of its poles—the better one, the most interesting one; or so we thought. To add the environment to our conceptualization of the world still leaves us trapped in a conceptualization of an anthropocentric world. We still focus on Society. We just start taking into account the ways in which human action conditions Nature.

“Thinking through the environment” (Rose et al., 2010) *should* be unsettling for sociology. It should lead us not to rethink but rather to fundamentally unthink (Wallerstein, 2001) what sociology has taken for granted for far too long. This exercise of unthinking Western modernity and its foundational epistemological and ontological assumptions leads to the radically relationist study of the multiple and heterogeneous interconnections between different life-elements of the world(s), neither of them *a priori* classifiable as belonging to “humanity” or “nature” but rather thus constituted through and along the very process of interrelation. As such, adding up Nature to Society (or Humanity, or Culture, or any equivalent) does not do. This position validates the reification of both terms and keeps them, as they have been for 500 years in slightly different ways, in relation *to* each other (Moore, 2016a,b). And, given human privilege *vis-à-vis* the non-human, Society's relation *to* Nature wrongly distributes agency, viewing it solely as a human capacity, thus turning nature into mere passivity. Even narratives on the Earth's revenge on humanity reinforce this insofar as in them nature's action is mostly re-action to the effects of what humanity—the only true actor in this story—does.

Unthinking what we know—including what we know about how we know—implies refusing to understand this issue in terms of humanity's relation *to* nature but rather conceptualizing it in terms of the space-time specific interrelations of different elements of the web of life. These are not in relation *to* each other, and much less are they in a binary relation in which one of the parts acts upon the other, the former's actions generating a set of consequences on the latter. These multiple life-elements enter into multiple space-time localized relations *with* each other, collectively establishing contingent, dynamic, and conflictual arrangements of human and extra-human beings and things—in Haraway's (2016, p. 34 *et passim*) formulation, composing “multispecies muddles.” In other words, through their collective practices they compose collectives; they collectively enact worlds.

In this essay we propose to unthink some of sociology's foundations of knowledge and practice. We do this by focusing on the Western modern dominant concept of nature, and particularly by discussing its transformation into the environment. The first section starts by looking at the Cartesian conception of the world in terms of Society/Nature, which necessarily subordinates the life-elements placed in the second term to those placed in the first. We then locate the Society/Nature divide within the wider Western modern dualistic logic, which is inherently totalizing and hierarchical. With this background, we argue that current mainstream sociological approaches to the study of human-nature relations have not sufficiently broke with its paradigmatic Western modern origins, making them unable to understand the multiplicity of interrelations between life-elements by which socioecological phenomena are enacted. The following section starts by looking at how nature is turned into the environment of Western modern humanity, which is an essential process for the latter's dominant approach to the government of human life. We then discuss how this is inextricable from capitalism insofar as it allows nature to be enacted as a series of commodities. Following this we argue that if the concept of the environment is inherently problematic, then environmental sociology has a foundational problem that it may be unable of solving. The section ends by contending that, due to these conceptual and practical problems, the environmental crisis is the wrong framing for the current Western modern crisis of enactment of socioecological ontologies and worlds.

There are far more sociological concepts and practices in need of unthinking. And much more could be said about nature and (its conversion into) the environment. Our position in this essay is quite humble and has no pretension to exhaustively discuss all that need to be unthought. We are merely pointing to some of the perplexities that have been bothering us in our common research, thus adding to the collective effort(s) of unthinking Western modern enactments of life with the hope that others will find this exercise relatable to their own intellectual and political concerns.

## Questioning Nature

The last 50 years have seen the emergence of many significant contributions to the exercise of unthinking Western modern enactments of the world(s) that inspires this essay: from the historical-philosophical *tours de force* of Foucault (1994, 2005a,b, 2012a), Kuhn (2009), or Feyerabend (1993), to science and technology studies (STS) and actor-network theory (ANT), passing through several schools of feminist and decolonial thought^5^. All of these have been listened to and developed by many other researchers, and we build our own work from and alongside them. Nonetheless, the critical exercise of unthinking and re-enacting is still far from being the norm for research carried out within sociology. This is undoubtedly the case of research on matters of human-nature relations.

Most sociological research on the general field of the relation of humanity to the rest of the cosmos has a firm footing on the Cartesian division of Nature and Society (or Culture, or any equivalent) (Descartes, 1982). As is the case of other fields on inquiry, research on “the environment” develops both through stances that are (to various degrees) critical of certain arrangements of practices characteristic of Western capitalist modernity and through positions which present—and, many times, believe—themselves as defending the axiological neutrality of science. Both of them are very much heterogeneous and this analytical partition is merely a shorthand. But most of these stances tend to implicitly validate and solidify the Social Contract argument of Hobbes (2002), Rousseau (2003), or Locke (2001), according to which the creation of any sort of civil and political—in essence, social—state is inherently dependent on the exit from the state of nature^6^. In this fashion, the sum of human beings, all of them exemplars of *ego cogito*, is withdrawn from the rest of the cosmos. All that is not humanity is thus transferred to one or more of the following categories: chaos, vital threat, landscape, romantic ideal, and/or resource reservoir.

No matter what category each of the constitutive parts of the non-human is put in—and the exact distribution varies dynamically according to space and time—it is thought of as being in relation to humanity. However, by definition, it is not part of humanity. Human and non-human (natural), their ontologies are different, even if the existence of each of them is ontologically dependent on the existence of the other. *Ego cogito* does not change his or her essence because of the action of the elements of the non-human^7^. He is *in* himself and *from* himself. In the same manner, nature's essence is unchangeable. The modern project of dominion of nature (Plumwood, 1993; Scott, 1998; Serres, 1998; Latour, 2010; Debaise et al., 2015) operates around the idea of taming nature, of molding it to humanity's wishes (or rather to the wishes of some members of humanity). In this fashion, Western modern human action is able to reshape nature's appearance, to cut down trees, to relocate animals and plants, to make water change its course, to hollow out nature by extracting what lies within it, to disrupt its homeostatic equilibriums. But this does not change nature's essence as Nature opposed to Human, as Nature in so far as it is non-Human. Nature's role as the Great Outside of the Human is not up for questioning. Paradoxically, it is assumed to remain (ontologically) the same even as it is made to change (geologically, geographically, biologically, etc.) by human action. It remains reality out-there, existing independently of how it is perceived, prior to statements made about it, in a definite and singular form (Law, 2004, p. 23 *et seq*.). It can be seen, interpreted, measured, classified, used, etc., precisely because of its ontological stability.

The Society/Nature divide is a fundamental foundation of Western modern thought patterns, which are inherently dualistic (Santos, 1991, p. 14 *et seq*.; Santos, 2006, p. 91 *et seq*.; Plumwood, 1993; Said, 2003; Castro-Gómez, 2005; Lander, 2009)^8^^,^^9^. There are many dichotomies that play a relevant role in Western modernity—Mind/Body, Self/Other, White/Non-white, Metropole/Colony, West/East, and so forth. But Society/Nature, alongside Subject/Object and Male/Female, are the fundamental modern dichotomies in reference to which all others work (Plumwood, 1993, p. 41 *et seq*.). Importantly, modern dichotomies are expressions of hierarchical relations, one of the terms being privileged and the other subaltern. As Plumwood (1993, p. 41 *et seq*.) argues, this hierarchical logic is where the command role played by the former three dichotomies becomes clear: the privileged term of every other dichotomy—Mind, Self, White, Metropole, West, etc.—tends to be seen as having characteristics associated with Masculinity, Society, and the active and rational Subject, whereas the subordinate term—Body, Other, Non-white, Colony, East, etc.—tends to be associated with Femininity, Nature, and the irrational (in the sense of being incapable of rational thought) and passive Object.

This hierarchical logic is clear in the dominant conceptualization of Society/Nature. The mythologem that is the state of nature is what one leaves in order to collectively create a social existence, the only one that truly matters, the one that, although imperfect, is far better that the alternative of nature—with its chaos, dirtiness, discomfort, aggression, etc. And we must keep in mind that, within this paradigm, nature is in fact the only alternative to society. It is the sole alternative because dichotomies are exercises in totality-as-way-of-ordering-reality, i.e., they are representations of a universe in which nothing can exist outside either one or the other of the two entities in relation, thus conceptually eliminating even the imagination, not to mention the *praxis*, of an existence unrelated to this logic (Plumwood, 1993; Santos, 2006, p. 91 *et seq*.).

Most sociological research on issues of the environment implicitly solidifies this philosophical stance. Uncritically taking for granted that there is something fundamentally different (i.e., better) in humanity (or society, or culture, or whatever else one chooses to call it), this heterogeneous field of inquiry tends to practice a “sociology of the social” (Latour, 2007) which, paradoxically, is extended from the social to its outside—without ever truly rearticulating the terms of their relationship. For Latour (2007, p. 160 *et passim*), a sociology of the social is doomed to be unsuccessful in its enterprise of understanding the uncertainty and dynamism of human life because it tries to explain the social by the social instead of focusing of the myriad processes of association of human and extra-human life-elements by which both “the social” and other realms of thought and practice are enacted. The practice of a sociology of the social to study issues considered to be outside the social creates an epistemic conflict: not only should the social be explained by the social but nature, for centuries understood in Western modern thought as explaining itself as much as society does (as long as science was able to progressively determine its/their laws of operation), now also is (at least in part) explainable by the social^10^. The passivity of nature characteristic of Western modern philosophy, opposed to the reflexive action of humans, is thus reinforced—even in narratives about the Earth's revenge on humanity because of the damage the latter inflicts upon it nature's action is re-action, making humanity into the true actor of the story insofar as without it no movement would have been made by the other term of the dichotomy.

A sociology of the social can do nothing but fail when trying to understand the heterogeneous interrelations of human and extra-human life-elements. It fails by design—albeit not reflexively so—because such a sociology is firmly grounded on the Society/Nature divide and, from this standpoint, multiplicity is not visible. If it cannot be seen, the myriad connections between different life-elements cannot be made into this sociology's central research topic. As such, the worldly relations explored by this field must be reduced to those that are understandable in terms of Society/Nature. In this manner, as a starting point, the world's life-elements are distributed into the categories of this dichotomy. When research starts, this has already been done, which leads to the placement of the elements of the two categories in relation *to* each other—fundamentally distinct, one of them acting over the other. If they are in relation *to* each other, they cannot be in relation *with* each other. This would presuppose that there are more than two elements in relation, it would presuppose that worlds are not yet taxonomically distributed into categories, leaving their life-elements relatively free to roam and communicate with one another with disregard for analytical borders. It would assume that these worlds are not only where these life-elements act and exist but also that they are the contingent and dynamic result of this very action and existence—a form of existence-as-action which can only be carried out by the efforts, work, and energy of space-time localized humans and extra-humans.

This leaves a sociology of the social with only one way of conceiving human-natural relations. If life-elements do not interact in ways that enact worlds—which, among other things, perform-contain contingent stabilizations of nature and society—, then, it is from within each of the two realms of Society and Nature that all things must emerge, eventually overflowing from one to the other. In other words, a sociology of the social can only conceive a world in which phenomena specific to one of these realms condition existence in the other. This is a cause and effect model of limited interrelation in which, generally, human action—the action of the Human—develops through human-specific processes that occur in the environment of Nature. There is no significant interplay in the generative process by which these phenomena start; they occur because humans do things with one another. But what they do together has such a magnitude that converts them into causes of—mainly damaging—natural processes (e.g., deforestation, emissions of greenhouse gases). Human actions emerge as causes of these phenomena, leading to a set of consequences which occur in the realm of Nature, depleting and degrading it. This can, eventually, come back to haunt us; but we alone caused it and the second level consequences—from human to nature back to human—by which natural phenomena with human causes damage Society do not make Nature into a true actor in this story. Granted, this cause and effect mode of thinking can be made into something complex, attuned to the idea that different human processes can combine themselves to cause one outcome and that the same human phenomenon can contribute to several environmental consequences. But this only works if the Society/Nature divide is accepted and validated. And even then this limited conception of action and interaction is inherently incapable of seeing much of the actions and interactions by which the worlds in which we—humans as well as others—live are collectively enacted.

Only by rejecting this dichotomy as something that exists *a priori*, as something that predates action(s), and by understanding it as the contingent and dynamic result of (both human and extra-human) action(s), can multiplicity be taken into account. The multiplicity of heterogeneous relations between different life-elements—human, animal, plant, or mineral—is what enables each and all of them to act and to enact different arrangements [or, in Moore's (2016b) term, “bundles”] of human and extra-human (Callon, 1986; Law, 2002, 2004; Mol, 2002; Latour, 2007, 2010; Haraway, 2016; Moore, 2016b). And the life-elements that are distributed into nature or society—which are not predetermined once and for all but rather are the object of historical and spatial conflicts, as black slaves and most women could attest—act in ways that make inappropriate the cause and effect thought models of sociology of the social's study of the environment. When different life-elements come together to act—both in peaceful and (mostly) in conflictual ways—, they are not *a priori* “nature” or “humanity” but are thus made through and along their collective actions. And the actions of different networks of human and extra-human life-elements constantly overflow each of the networks that originally performs them to reach other such networks, creating multiple flows of mutual communication between what is, at a certain time and in a certain place, constructed as social and natural.

Given these shortcomings, a sociology of the social provides an inadequate framing for the understanding of the myriad relations between humanity and nature. No matter how critical it may be, research developed within this paradigm falls back into a form of Western modern reification of both Society (or Humanity, etc.) and Nature. In other words, it starts from an implicit decision to distribute the world's life-elements into these two—and only these two—categories and then proceeds to ascribe them two ontologically incommensurable essences. It is only if this conception and practice of sociology is rejected that it is possible to comprehend the myriad interrelations between (human and extra-human) life-elements. In order to move beyond this paradigm, after having started to explore the conversion of the extra-human into Nature, we now continue the discussion by looking at the enactment of Nature as the environment.

## Nature Becomes the Environment of the Human

Perhaps the main shortcoming of the extension of a sociology of the social to the study of the relations between human and extra-human is immediately visible in the expression “environmental sociology.” What does this sociology deal with? The environment of something that does not belong to it. It deals with reified nature, understood as the outside that is all around equally reified humanity^11^. As Serres puts it, “the word *environment*, commonly used in this context (…) [,] assumes that we humans are at the center of a system of nature” (Serres, 1998, p. 33).

Such a sociology most definitely does not study the dynamic and heterogeneous interrelations between different things of and in the world, it does not highlight how these temporally and spatially specific interconnections between human and extra-human life-elements are precisely the processes by which worlds and those who live in them are collectively enacted. In short, it does not address the various forms of creating certain space-time specific arrangements of life, i.e., of creating contingent and precarious realities and of distributing their component elements in them by processes of categorization as human and other-than-human (Law, 2004; Latour, 2007, 2010; Debaise et al., 2015; Haraway, 2016; Moore, 2016b).

Instead, sociology starts from the positive exception of the Human. In Western modernity, given the hierarchical relation of the terms of the duality that is Society/Nature, the Human is not only outside of the non-human; it is above it. It is epistemologically, ontologically, and morally superior to Nature^12^. Nature appears in relation *to*—and never *with*—humanity, merely as the *milieu* of its life chances, as the resource reservoir from which humanity derives “natural resources” and, depending on space and time, as *locus* and *arche* of potential threats to its life.

As Foucault made clear, the emergence of a biopolitical rationality of government^13^ in eighteenth century Europe elevated the concept-*praxis* of (human) population to the role of central subject-object of intervention (Foucault, 1980, 1994, 2006, 2009, 2010). Around this period, the exercise of power took as its main preoccupation the protection of the human life of the collective that is population, aiming to increase its life opportunities by guaranteeing that its behavior did not deviate from statistical-scientific normality in ways that endangered it. The consolidation of industrial capitalism, the maintenance of colonial residents and administrations, and military strength-in-numbers within (as well as outside) Europe, all required large quantities of relatively healthy human beings. In order to meet this requirement of protection of human life (at least of that human life which power-knowledge conflicts lead to be placed into categories of the Human), governmental interventions became more effective by indirectly guiding these human collectives instead of directly prescribing and adjusting their conducts. As such, governmental exercises assumed the form of interventions on the *milieu*, the environment in which populations lived, aiming to change the manner in which collective phenomena were shaped by changing the conditions which framed the possibilities for each unit of the population to act (Foucault, 2009, p. 29 *et seq*.). The underlying logic is simple to explain, even if the processes by which it is enacted are very much complex. Want to decrease mortality rates and increase the general health of the inhabitants of a certain city? Don't prohibit individual behaviors that make people sick, like unsanitary eating or hygienic habits. Don't threaten individuals with the strong arm of the law in order to stop them from doing what has been scientifically discovered to be harmful for them. Instead, construct and maintain centrally regulated urban sewage systems, create a process of regular garbage collection, or lower taxes on food rich in protein and vitamins.

Granted, Foucault's focus is not on the environment understood as Nature—even if the phenomena which affect a human population's life are both “natural” and “social”: laws, commerce, traditions, or taxation, as well as food, climate, or disease (Foucault, 2009). Furthermore, his periodization of Western modern intervention on the environment in order to govern life is off by some 200 years (McBrien, 2016; Moore, 2016b; Parenti, 2016). But his insight on the central role played by the environment of humanity in Western modern governmental exercises must not be downplayed^14^. If one dates the start of Western modernity to the transatlantic colonial arrival of 1492 (Wallerstein, 1993, 2004; Dussel, 1995; Mignolo, 1995, 2000; Lander, 2005; Quijano, 2007; McBrien, 2016; Moore, 2016b), it becomes clear that humanity (at least that part of humanity which arrived on American shores and its descendants) has since then constructed the extra-human as being up for grabs.

This is the geohistorical^15^ moment of the start of the Western modern logic of “mastery and possession” of nature—“the master words launched by Descartes at the dawn of the scientific and technological age, when our Western reason went off to conquer the universe” (Serres, 1998, p. 32). As Dussel (1995) argues, the *ego cogito* was historically preceded by the *ego conquiro*, the Human who, having arrived outside of Europe, immediately defined the world of the non-human as existing solely for his benefit. This was the premise behind the definition of the “New World”—and, with it, of the totality of the non-human—as *terra nullius*, mere nature without humans which could for this motive be freely appropriated by humanity (Johnston and Lawson, 2005, pp. 364–365; Mignolo, 1995, p. 260 *et passim*; Plumwood, 1993, p. 111, pp. 161–163; Wolfe, 2006). The process by which large portions of humanity were relegated to categories of Nature-outside-humanity was simply the necessary condition of this operation of humanity's mastery and possession of the world of the non-human. So, a double disqualification is at work in Western modern paradigm's conception of Society/Nature: on the one hand, to the Human belongs the world, subordinating nature to humanity; on the other hand, the Human is reduced to the part of humanity which in fact masters and possesses nature, *de facto* and/or *de jure* de-humanizing most human beings (in various ways), thus disqualifying them as they are placed in the concomitantly disqualified space (and time^16^) of nature (Dussel, 1995; Moore, 2016b, pp. 78–79 *et passim*).

This is the paradigm in which environmental sociology moves itself. What it sees and how it sees it are strongly conditioned by the manner in which nature is transformed into the environment of humanity. As environment, nature is enacted as the mere surroundings of the Human, the latter existing at the center of an undeniably anthropocentric cosmos. Since this is a Western modern cosmos, *Anthropos* is clearly defined. He is *ego cogito* but that is not all that he is. *Anthropos* at the center of the universe, *Anthropos* for whom the universe exists, is also a capitalist being—perhaps this is what he primarily is, as the world-ecology school of thought argues by defending the “Capitalocene” as the most precise concept to encapsulate the current geohistorical era (Moore, 2016a,b). As such, within the Western modern paradigm, nature is enacted as the environment of *homo oeconomicus*.

As the environment of modern *homines oeconomicae*, nature—or, to be more precise, all supposedly non-human elements of the anthropocentric world—are made into resources to be conquered, dominated, and appropriated (Serres, 1998; Moore, 2016b). The reification of the extra-human as Nature is the first step of a process by which all discrete units of this Nature are enacted as potential resources to be used and depleted with all the might and the right the Human confers upon himself at the expense of all other beings and things. The environment of humanity is humanity's reservoir of potential resources. In this manner, nature-as-environment loses any meaning in itself and all of its potential significance derives from the use Western modern capitalist humanity gives it. Its lack of meaning outside of Western modern capitalist standards makes reified nature into an entity whose discrete units, both those that are known and those that might be known, are made into things-as-potential-commodities. It is not the case of the non-human being immediately enacted as commodity. Rather, it is enacted as something that, in itself, is nothing besides a collection of smaller things, each one of them potentially commodifiable^17^. In other words, nature-as-environment has no meaning besides that which Western modern capitalism is able of giving it and each of its components, and this societal model is only able to give meaning to commodities (or, to put it more precisely, it is only capable of ascribing meaning to something by commodifying it). At any given time, the environment has some discreet units that are not commodified, as well as others that are. According to the space-time specific necessities of capitalist modernity, the life-elements that are categorized as any form of Nature are brought from the field of potential commodity to that of actual commodity (and vice versa), thus expanding the total field of capitalist commodification of the non-human world^18^.

This *modus operandi* of commodification by grabbing parts of the environment and re-signifying (i.e., re-enacting) them as things with mercantile value transforms these life-elements into “fictitious commodities.” For Polanyi (2001, pp. 71–80 *et passim*), a process of commodification has a “fictitious” character when it ascribes market value to things that were not produced with the explicit intention of being sold as merchandise. Thus, the commodification of such things de-signifies them insofar as the market is fundamentally incapable of exhausting their total meaning. In other words, they are far more than something with market value and to transform them into commodities is to reduce all of their cultural meaning to market criteria, which makes them into elements of the world whose total significance capitalism in not able to grasp, even though it is very much capable of using and abusing them. Polanyi's foremost examples of such “fictitious commodities” are money, labor, and land—the last of which he describes as “the natural surroundings in which [society] exists,” making “land [into] only another name for nature” (Polanyi, 2001, p. 75).

Polanyi's discussion of “fictitious commodities” is framed in epistemological and ontological terms that are conflictual with the position we espouse in this essay. He is clearly conceptualizing commodification through the lenses of Society/Nature, reifying land-as-environment, as well as essentializing the remaining “fictitious commodities,” as is apparent when he writes that “labor, land, and money are obviously *not* commodities: the postulate that anything that is bought and sold must have been produced for sale is emphatically untrue in regard to them. In other words, according to the empirical definition of a commodity they are not commodities. (…) None of them is produced for sale. The commodity description of labor, land, and money is entirely fictitious” (Polanyi, 2001, pp. 75–76). In this sense, when he writes that “land is only another name for nature”, he immediately adds that this nature “is not produced by man” (Polanyi, 2001, p. 75).

Following Moore (2016b)—and taking a cue from a staple position in STS and ANT—, we can argue that, in Western capitalist modernity, everything that is commodified is “originally” produced as a commodity (by being grabbed from the resource reservoir that is the environment and enacted as a commodity). The point is that there is no such thing as Nature out-there with an original essence that puts it outside the collective action of human-and-extra-human arrangements by which space-time specific ontologies and worlds are enacted—some of them as commodities. The process of “originally” producing something as a commodity is precisely this enactment.

Nonetheless, Polanyi's insight is valuable in two ways. On the one hand, it makes clear that commodification is inherently incapable of exhausting the potential meanings—the potential life—of the elements of the world(s) which are commodified (i.e., other enactments of these life-elements are possible and none has the totalizing capacity of exhausting all that any of them might be made to be). On the other hand, Polanyi's argument highlights that the peculiar market-based enactment of some life-elements as commodities is inherently damaging, both to them and to the world(s) to which they belong to (i.e., which they, together with other life-elements, compose).

In this derivatively polanyian sense, the process of turning non-human elements of the world(s) into a series of “fictitious commodities”—i.e., into resources for human production and consumption, into things that exist solely to guarantee the life of the Human^19^—is at the very core of Western modernity. This societal model exists because the extra-human is turned (reified) into Nature, which in turn is transformed into the environment of the Human and dealt with (i.e., enacted) as a reservoir of potential commodities. It is this particular kind of commodification that enables the typically Western modern capitalist human modes of action and existence that do not reflexively take into account the manner in which different networks of human and extra-human life-elements act together to enact certain types of worldly arrangements (i.e., certain types of worlds). In other words, this sequential process starts with the reification of the non-human, follows to its conversion into the Great Outside, there solely for the use of the Human, then fragments this environment into discreet units, and lastly picks and chooses which units will be commodified in a given space and time. It is this process that enables the kinds of careless human action and existence that disregard the wellbeing of the extra-human, *in extremis* disregarding the very condition of possibility of its existence. Given that the different life-elements of the world(s) do not adjust themselves willingly to Western modern fragmentation of reality—or, to be more precise, Western modern's enactment of fragmented realities—, the forms of human action and existence that are made possible by the sequential process of reification of the extra-human are both genocidal and suicidal. The practical symbiosis of human and extra-human life-elements of space-time specific networks, symbolically denied in Western modernity, implies that the uncaring disregard that leads to the extermination of the extra-human also describes a suicidal operation by which the Human disregards its own conditions of possibility, its own conditions of a future, of any future^20^.

If this is the “environment” in “environmental sociology,” then, this field of inquiry has a problem. Not one it can discard or correct but something more profound, which marks its very core, making “environmental sociology” inextricably linked to this enactment of the environment. As such, the only way of successfully facing this problem is to unthink the core concept of the environment. But given the intimate connection of this concept to the field of sociology that studies it, to unthink the environment is to leave environmental sociology behind in direction to a conception and a practice of sociology that has discarded the Society/Nature divide and taken as its focus the myriad interrelations between different life-elements of the world(s) by which contingent, precarious, and dynamic “multispecies muddles” (Haraway, 2016, p. 34 *et passim*) are enacted. This would be a sociology which, while being attentive of human peculiarities, would not presume humanity to be the only peculiar entity in the cosmos and would rather assume that giving due attention to the interconnectedness of human and extra-human life-elements of specific space-times is fundamental to the adequate understanding of the phenomena it deals with. In other words, in order to make environmental sociology relevant at a politically and intellectually fundamental level, it must be unmade and reforged into something very different from what it was and still predominantly is.

If the environment is part of what must be unthought, a field of inquiry that takes it to be its core concept—or at least one of its core concepts—cannot frame the right questions for the right issues, thus making it unable of providing hypotheses and coordinates for action which might be used to face the problems at hand. It is unable of providing these hypotheses and coordinates because it is not looking at the phenomena that need to be looked at. The prime example of this is perhaps the focus on environmental problems, many times conceptualized as an (the?) environmental crisis. There can only be an environmental crisis if the extra-human is reduced to the Great Outside of the Human. Only in this paradigm does human action damage what is fundamentally other-than-human, creating a sustainability problem.

Within this paradigm, many are the solutions proposed to this unsustainability of the life of the Human. These tend to be framed in the general terms of greening capitalism, of making sure that Western modern capitalism survives by trying to reduce the rate of world(s)-destruction, thusly guaranteeing the eternal reproduction both of this societal model and of its inherent destruction of worlds, including itself. The main approach to this is technocratic (Crist, 2016; Hartley, 2016), appearing in the form of proposals to reforest critical areas of the planet; of geoengineering projects (Altvater, 2016); of attempts to reduce greenhouse gases emissions by replacing fossil fuels with renewable energies or through regulated market trade of carbon credits (Vossole, 2013); and so forth. At best, these are all short-term palliatives aiming to minimize—but not to fundamentally combat—climate change and other “environmental” problems. All of these solutions are doomed to fail simply because what is problematic is Western modern capitalism itself—within which both the damages and the solutions are being enacted (Serres, 1998; Moore, 2016b)^21^.

Environmental sociology is, at most, a very minor player in this game of climate/environmental-palliative prescription^22^. But it does share with this technocratic approach the same core concepts of the environment and the environmental crisis, thus reflecting many of the same shortcomings that are apparent in policy-making, engineering, economics, the natural sciences, and other technocratic fields. So maybe it is time to leave environmental sociology behind. In the face of contemporary threats to planetary life, it is increasingly urgent to move on to the radical relationism of space-time specific multiple and heterogeneous arrangements of different life-elements of the world(s), both human and extra-human. If we start making this movement, the environmental crisis is shown as fundamentally inadequate as a problem with which we should concern ourselves. It is shown to be a life-and-death-enactment fraught with the same symbolical and material problems that have marked Western modernity since its beginning—the very same problems that have brought about a state of affairs in which very real dangers are upon us. Since the environment is a severely limited, blind, and extinction-prone way of enacting the extra-human, the environmental crisis is the wrong framing for these dangers.

None of this means that all is well; far from it. Although we distance ourselves from certain discourses about the current and/or inevitably coming planetary (i.e., “environmental”) catastrophe^23^, here, we stand with Latour (2011): one should give due attention to the Apocalypse brought about through poorly enacted realities of human and extra-human entanglement; it is not because the End has been repeatedly announced throughout history and never came that one should blind him or herself to the fact that profound and rapid changes to the biosphere are verifiable and very likely to increase in the near future, making the Apocalypse a significant material possibility—at least for humanity, but we can be sure that if we go down we will be taking others with us.

There is a vital crisis—literally a crisis of vitality, a crisis of life enactments—but this is a crisis of world(s)-building. It is a crisis of Western modernity and of the types of life-realities that are possible (and impossible) to enact within its boundaries. It is a crisis of a societal model that, as Marx (1975) reminded us, is based on the alienation of humanity from nature, making the latter into a mere means of guaranteeing human life—which is inextricable from the alienation of each human being from what he or she produces, from his or her own self, and from other human beings^24^. This changes the problems we—both human and extra-human—have to face, making it inevitable to conclude that only through revolutionary change^25^ of the Western modern capitalist societal model could the world(s)-building crisis be unmade—even if its consequences will very likely shape the conditions of possibility for most, if not all, future enactments of human-with-extra-human arrangements of life.

## (Definitively Not) Conclusions: Unthinking From the Margins

How can we leave the world(s)-building crisis behind? How can Western modern problematic enactments of the web of life be successfully unmade and remade in ways that do not oppress the world(s)' life-elements, both human and extra-human? Unthinking the epistemic foundations of both Western modernity and of its predominant forms of knowledge, including the social sciences, is the first step for the much-needed reenactment of life. One of the things that this process leads to is to the reforging of environmental sociology into something quite different from what it has been. It leads to dropping the environment from a sociology that concerns itself with the multiple, heterogeneous, space-time specific relations of life-elements by which humanity and nature are contingently made and remade.

But how can this be achieved? Any answer to this question is fraught with the pitfalls of *hubris*. Aiming to provide definite answers to similar questions is a very Western modern stance. It is, without a doubt, possible to attempt to do so—but only at the risk of replicating the very paradigm that created the problems discussed in this essay, as well as many others. We do not have any such proposal to close what has been said. We cannot have it because what has been said is entirely open-ended. And since life is always locally enacted in particular places and times, by particular networks of human and extra-human elements, the problems of life can only be—precariously—dealt with by each of the multitudes that are implicated in its enactments. Answers for problems related to the enactments of life can—and quite likely need to—be inter-locally coordinated, but no one locality or actor is able of providing them for the others. All that we dare to put forward are tentative sensibilities and intuitions.

By their inter-local contingent and conflictual coordination(s), the multiple processes of life-reenactment that are needed in order to overcome life-and-death issues such as changes to the biosphere, deforestation, or the extinction of entire species, are inherently revolutionary. And revolutions are arduous things to make—especially when, like what is at stake in these cases of life-enactments, they cannot be made once and for all.

One cannot leave Western modernity by establishing something else in an instant. It is not possible to enact what one cannot imagine and our—individual as well as collective—imaginations are severely—albeit not completely—limited by Western modern habits of thought and practice. Given this, any revolution of Western modern forms of enacting life can only be done from within Western modernity itself. Fortunately, Western modernity is not homogeneous. It is a succession of life-enactments that have manifold forms, although they share some fundamental (i.e., paradigmatic) assumptions.

As Law and Lien (2012a) remind us, Western modernity enacts the Society/Culture divide both coherently and non-coherently. This societal model has an undeniable will-to-singularity. It attempts to construct singular ontologies of both Society and Nature that are valid everywhere. But, at the same time and with the same relative importance, even within Western modernity there are multiple enactments of society and nature. Each specific space-time makes and remakes their own version of both. This does not annul the will-to-singularity but rather makes unicity inherently dependent on multiplicity.

This is a relevant opening. It allows for revolution from within. It allows for the exploration of the multiplicity, contingency, and ambivalence that are intrinsic to Western modernity as a possible way of reframing what this societal model has tried to solidify. In this manner, it might be possible to shake things up just enough to turn solids into fluids, to agitate life-elements just enough to make it possible for them to connect with each other in different, non-oppressive and non-extinction-prone forms. Unthinking what and how we know about the elements of life is one of the fundamental processes by which Western modernity can be productively and revolutionarily shaken through its non-coherences.

It is much more likely that this can be done not from the center(s) of Western modernity but from its margins. By definition, these margins are not outside of this societal model and of its predominant ways of knowing. But these are the places where Western modernity, through its non-coherences, comes into direct contact with the possibility of something other than itself. It is from here that a transformative exercise of “border thinking” (Mignolo, 2000)—or, rather, border unthinking—can be carried out. These margins are the places where ontologies and realities are not-quite-made, where they are almost-enacted, where they start to be performed by local networks of life-elements but are then interrupted and discarded^26^. But even though they are not fully made, they make a statement about the potentiality of other ways of enacting the web of life. And even when these other forms of enacting ontologies of and relations between “nature,” “humanity,” and other categories, are dropped due to their impracticality or their high costs, their potentiality remains.

A sociology that deals with the multiple enactments of the web of life—and definitively not one that is “environmental”—can contribute to this border unthinking. It can do so by looking at how the world(s) can be, not in a metaphysical sense, but by exploring empirically partially enacted potentialities. It can do so by looking for those ontologies of nature, humanity, and so forth, that, although not completely enacted, are perceived and whose making is started by local actors only to then be interrupted and discarded for a myriad of reasons. It is from what can be (made to be) that it might be possible to productively fracture Western modernity's crisis of world(s)-building in direction to a multiplicity of space-time localized socioecological enactments that do not subjugate life, whether it is contingently distributed and performed as human or as extra-human.

## Author Contributions

JA and FA contributed to the development of this essay's arguments, as well as to the writing and revision of the submitted manuscript.

### Conflict of Interest Statement

The authors declare that the research was conducted in the absence of any commercial or financial relationships that could be construed as a potential conflict of interest.

## Footnotes

^1^We are borrowing the formulation of Rose et al. (2010): “Thinking through the environment, unsettling the Humanities.”

^2^This article's arguments focus on the case of environmental sociology. The fact that we are both sociologists is not irrelevant to this choice as it makes sociology the discipline whose limits and potentialities we know best. Nevertheless, the issues discussed are of broader paradigmatic consequence to the (heterogeneous) whole of Western modernity. As such, we are inclined to believe that many of what is said might be pertinent to discussions held in other fields of knowledge that still regard themselves as studying “environmental” phenomena.

^3^We use the term “life-elements” to refer to all entities of the world(s) that, in one way or another, contribute to the collective enactment(s) of life. These are both biologically living elements and all the other things of the world(s) that, together with the former, make up what Moore (2016a,b) calls the “web of life.”

^4^See Moore (2016a,b) for a critique of this “green arithmetic,” “the idea that our histories may be considered and narrated by adding up Humanity (or Society) and Nature, or even Capitalism plus Nature. (…) [S]uch dualisms are part of the problem—they are fundamental to the thinking that as brought the biosphere to its present transition toward a less habitable world. (…) [T]he categories of “Society” and “Nature”—Society without nature, Nature without humans—are part of the problem, intellectually and politically” (Moore, 2016a, p. 2).

^5^There are too many references to quote for any of these (internally very much heterogeneous) lines of work. We merely highlight some of those we consider to be especially relevant for this exercise of unthinking. For STS and ANT, see Callon (1986), Latour (2007, 2010), Latour and Callon (1981), Law (2002, 2004), and Mol (2002). Haraway (1991) and Plumwood (1993) present two feminist positions that are particularly relevant for issues discussed in this essay. For the case of decolonial thought, see Dussel (1995); Mignolo (1995, 2000); Lander (2005), Said (2003), and Quijano (2007).

^6^Hobbes, Rousseau, and Locke had fundamental divergences on several topics, which cannot be discussed here. Nonetheless, they shared the general argument mentioned.

^7^He might modify his lifestyle as a response to altered environmental conditions, but this is another matter altogether.

^8^One should bear in mind that the idea that there is a difference between mind and body, as well as between humanity and nature, is not an idiosyncrasy of Western capitalist modernity (Plumwood, 1993, pp. 69–103 *et passim*). However, as Moore puts it, “capitalism was the first civilization to organize itself on this basis” (Moore, 2016b, p. 84).

^9^Law and Lien (2012a) remind us that nature and society (or culture) are paradoxically both coherent and non-coherent in Western modernity. On the one hand, there is an undeniable will to make them singular—only one Nature and one Society, which are the same everywhere. On the other hand, since both nature and society are enacted by the practices of actor-networks that are bound to particular places and temporalities, each local network enacts—slightly or pronouncedly—different ontologies of nature and society, and therefore different forms of their relationship. But even if no two enactments of Society/Nature are the same, at any given space-time, the Western modern dualism of Society/Nature that is being enacted tends to be (re)presented as singular (i.e., as *the* form of Society/Nature). And, since these different enactments coordinate themselves in various ways, they converge (dynamically and contingently) to form one ontology of Society and one ontology of Nature—that is always dependent on their multiplicity. In this manner, much like Bauman (1989, 1991) has shown, Western modernity's inherent ambivalence emerges as its defining characteristic, making ontological multiplicity not that which negates ontological unity but rather that upon which this unity is made (Law, 2002, 2004; Mol, 2002; Law and Lien, 2012a,b). We will return to this in the conclusion of this essay.

^10^Nature is now *at least in part* but not *entirely* explained by the social. The extension of a sociology of the social to the study of phenomena historically considered to be outside of society presupposes that natural phenomena are conditioned by human action. As such, what happens in society changes nature—geographically, geologically, biologically, even if not ontologically—, making nature only understandable if society is taken into account. But this logic does not negate scientific specialization, i.e., it does not deny that there are natural phenomena that can only be understood by the natural sciences—even if even the latter have increasingly started to take into account a (mostly homogenized and abstract) role played by Humanity in the shaping of the phenomena they study.

^11^Although we are discussing the specific case of environmental sociology, other environmental social sciences deal with the non-human in a generally similar fashion: they accept and solidify the fracture between Society and Nature, they grant a privilege to the Human, and they contribute to the transformation of nature into mere surrounding of *ego cogito*. Different social sciences have historically approached the environment in different ways. But, insofar as their practitioners regard what they study as the “environment,” these social sciences also share an epistemic positioning. A specific social science, sociology included, can have practitioners focused on understanding the socioecological enactment of worlds, as well as practitioners who study the human (or social, etc.) dimensions of “environmental” phenomena. The latter work in the same *episteme* as environmental sociologists, even if the former might not. Since this is a Western modern conception, in general terms, the natural sciences subscribe to the same conception of nature-as-the-environment-of-the-Human, albeit their focus is on the other side of the divide (with some researchers trying to bridge it without unmaking it, much like what happens in the social sciences).

^12^The Human is morally superior to Nature insofar as ethics is defined solely as an affair of humanity. Nature is not less moral, it is not immoral, it simply is—and in that pure existence, it is amoral in reference to the Human who thus becomes the only potentially moral entity.

^13^“Government” is understood by Foucault (1983, 2009, 2010, 2012b) as the “conduction of conducts,” which always occurs within the framework of a given governmentality, i.e., a certain “art of government” or “rationality of government.”

^14^See Parenti (2016, pp. 170–171 *et passim*) for a theoretical framing of Foucault's biopolitical logic of government through the *milieu* in terms that make it possible to mobilize his thought in the study of the multiple interrelations between human and extra-human life-elements. Parenti does this by highlighting the dimension of biopower which deals with the enactment of non-human nature(s) as a way of enacting specific human arrangements—which, given the necessary interconnections between human and extra-human implicated in these power-knowledge exercises, by definition makes them into specific human-and-extra-human arrangements. He calls this dimension of biopower “geopower”: “if biopower is about harnessing, channeling, enhancing, and deploying the powers of bodies at the scale of territorially defined populations, then *geopower* is similarly the statecraft and technologies of power that make territory and the biosphere accessible, legible, knowable, and utilizable” (Parenti, 2016, p. 171). The extension of Foucault's work to issues of human-and-extra-human enactments of collective life is being carried out by the environmentality (i.e., the environmental governmentality) school of thought. A brief exposition of environmentality's general stance can be found in Malette (2011).

^15^According to Moore, the multiple interconnections between human and extra-human life-elements through which material-symbolical worlds are enacted makes geology a fundamentally historical phenomenon. As he puts it, “the co-produced character of resource production, unfolding through the human/extra-human nexus,” which he names “the *oikeios*,” turns geology into “geohistory”: “Geology, in other words, becomes *geohistory* through definite relations of power and production; these definite relations are geographical, which is to say they are not relations between humans alone” (Moore, 2016b, p. 95).

^16^One of the upmost indicators of this disqualification is the representation of humans as existing at the head of the arrow of time, always moving forward through multidimensional-albeit-linear progress, whereas non-humans, in the state of nature, are immobilized—or at the very least very much slowed down—in a time of very little value by Western modern standards. See Fabian (2014) for a framing of this logic in terms of what he calls the “denial of coevalness.”

^17^This applies to human beings reified as part of Nature as well as to extra-human life-elements, i.e., to unpaid domestic labor developed in the *oikos* for the reproduction of human biological life as it turned into wage-labor as well as to fossil fuel reserves as they turned into one modern capitalism's main energy sources.

^18^See Moore (2016b) for a discussion of how capitalism functions by making a zone of exploitation of paid work-energy dependent on a zone of appropriation of non-paid human and extra-human work-energy. Life-elements are never placed in one of these zones once and for all but rather are moved from one to the other according to capitalism's space-time specific needs and capabilities.

^19^The life of the Human should be understood as a specific manner of living symbolically valued in Western modernity.

^20^See McBrien (2016) for a discussion of capitalism as a world-ecology inextricably linked to extinction, which the author frames in terms of the necrotic properties of capitalism. According to him, “capitalism was born from extinction, and from capital, extinction has flowed” (McBrien, 2016, p. 116). Capitalism is not only a productive system; its productive process is inherently based on destruction and death. If capitalism is a necrotic socioecological system, then, it is simultaneously genocidal and suicidal. Capital accumulation is only possible through the conversion of life into death—into resources to be depleted or into things to be annihilated because they stand in the way of these resources. In this manner, “extinction is both the immediate success and ultimate failure of the real subsumption of the earth by capital; the ecology of capital is constructed through attempted erasure of existing ecologies—ecologies that include humans” (McBrien, 2016, p. 117). This logic highlights the inseparable link of genocide and suicide in capital's necrosis insofar as it leads to a increasing production of negative value: if capital needs nature to appropriate and extinguish in order to generate value, then, the very process of capital accumulation decreases the part of nature that is available to be thusly appropriated, symmetrically increasing forms of nature that are hostile to this accumulation and cannot be incorporated into or avoided by this *modus operandi* in the *longue durée* (e.g., toxic waste, garbage, greenhouse gases). See also Moore (2016b) on the extinction of Cheap Nature by capital accumulation.

^21^We do not intend to reduce all proposals to deal with the environmental crisis to their technocratic variations. There are other types of proposals, namely those emerging from the heterogeneous schools of environmentalism. Their discussion is beyond this essay. Nevertheless, it can be argued that most of them operate in the same epistemological and ontological framework of scientific and policy-related technocracy, and thus share the many of the same problems. Sometimes the solutions they propose are dependent on the same technologies as scientific and policy-related technocracy. But even when they are framed around notions of “going back to nature,” of living in harmony with a Nature that exist out-there, undisturbed by human action, these solutions are firmly rooted in Society/Nature. While seeking to denounce Nature's degradation by Society, both entities are reified and solidly placed in relation *to* each other—and a relation in which humanity is the only true actor to be found among the multiple life-elements of the cosmos. The will to leave the environment undisturbed does so in more ways than one, making it impossible to leave the environment behind and move on to space-time specific enactments of worlds through the myriad interrelations of life-elements.

^22^In general, all environmental social sciences are minor players in this game, with the exceptions of (behavioral) psychology and (neoclassic) economics, which have a relevant role in environmental policy making (Shove, 2010). Nonetheless, even economics and psychology have not shifted the dominance of the natural sciences in this field.

^23^See McBrien (2016) and Haraway (2016) for a critical discussion of “catastrophism” and of how mainstream narratives on the Anthropocene reinforce this worldview.

^24^See also Moore (2016a, p. 86 *et passim*).

^25^Having just quoted Marx, the meaning of revolution could be misconstrued by some readers. We are not *a priori* framing it in any way, neither in terms of process nor in terms of teleology. By definition, the fundamental transformation of a societal model is revolutionary. And, also by definition, fundamental transformations are violent—sometimes physically, but always epistemologically and ontologically, and thus materially. But the specific character of such violent actions can only be defined by the actors who collectively develop the multiple space-time localized practices by which such transformations are brought about. Taking an example from decolonial historical processes, the “non-violent” Gandhi-model (Gandhi, 2006) of revolution is not necessarily less violent than the Fanon-model (Fanon, 2001, 2008), although they are carried out by very different sets of actors-elements and practices. The inherent violence of these processes is profoundly variable both in scope and in kind, and only during their development can it be decided and classified in any way. Analytically and politically, physical intra- and inter-species overt aggression is only one of the many forms revolutionary violence can assume.

^26^See Law and Lien (2012b) for an empirical discussion of these not-quite-enacted ontologies in the case of salmon farming.
